# The Epidemiology of Hospitalization for Pneumonia in Children under Five in the Rural Western Region of Nepal: A Descriptive Study

**DOI:** 10.1371/journal.pone.0071311

**Published:** 2013-08-07

**Authors:** Amrit Banstola, Ashik Banstola

**Affiliations:** 1 Department of Public Health, School of Health and Allied Sciences, Pokhara University, Kaski, Nepal; 2 Department of Pharmacy, Valley College of Science and Technology, Kathmandu, Nepal; Aga Khan University, Pakistan

## Abstract

Pneumonia is one of the major public health problems in children under five years of age. The aim of this study was to analyze the time, place, and characteristics of the distribution of pneumonia in hospitalized children under five years of age at the Dhaulagiri Zonal Hospital (DZH) in Nepal. A descriptive cross-sectional study was carried out at DZH from July 16, 2008 to August 17, 2011 for hospitalized children under five years of age and diagnosed with pneumonia. The main bacterial cause of pneumonia was *Streptococcus pneumoniae* and the main viral cause was Respiratory Syntical Virus (RSV). The majority of children admitted for treatment of pneumonia were males (60%), from upper class ethnic groups, and common among those aged 29 days to one year (49.1% of overall pneumonia cases). Data from this study show that pneumonia episodes in DZH occurred throughout the year with a sharp increase in the occurrence at the end of August to September. More cases were recorded during the rainy seasons and winter months in all three study years. The cases were from households most concentrated in Baglung municipality where the hospital is located. Pneumonia was found in higher proportions among hospitalized male children, those aged 29 days to one year, and in upper ethnic groups, during the rainy seasons and in winter months, and among local populations near the hospital in the rural western region of Nepal. Strengthening community-based case management, prevention strategies, and health care delivery system would help reduce pneumonia cases and the overall burden associated with it.

## Introduction

Pneumonia is one of the major public health problems in children under five years of age. It is an infection of lungs and most commonly caused by viruses or bacteria and spreads by direct contact with infected people [1]. The risk factors include poverty, lack of measles immunization, indoor air pollution, overcrowding, malnutrition/poor nutritional practices, lack of exclusive breastfeeding, and low birth weight [2], [3].

Pneumonia kills more children under five years of age than any other illness in the world [4]. Globally, every year, it kills an estimated 1.2 million children under five, making up 18% of all deaths of this age group [5]. According to World Health Organization (WHO) [6], “more than 150 million episodes of pneumonia occur every year among children under five in developing countries, accounting for more than 95% of all new cases worldwide”. The estimated incidence of pneumonia in children under five in the South-East Asia is 0.36 episodes per child, per year [2]. In Nepal, according to the recent annual report of Department of Health Services (DoHS), Ministry of Health and Population (MoHP), out of total 2,752,266 ARI cases among children under five, 822,352 (30%) were reported as pneumonia from July 17, 2010 to July 16, 2011 [7]. The report further states that the incidence of pneumonia per thousand under five year children was 246.

Dhaulagiri Zonal Hospital (DZH), a public hospital located in Baglung (a rural district of western Nepal) is a major healthcare provider and a referral center for the other hospitals/health facilities of Baglung and the surrounding districts (Parbat, Myagdi, Mustang). The Baglung district lies between 28°16′ northern latitude and 83°36′ eastern longitude and is at an elevation of 3,350 feet above sea level. The annual average rainfall of the district is 2200 ml and a minimum rainfall is about 10.9 ml. Rainfall is heavily affected by the Monsoon and most of it occurs during the months of June through September. Rest of the year the district is mostly dry and sunny. The temperature ranges from a minimum of 6.5°C to a maximum of 37.5°C.

Over the past few years, several attempts have been made to raise the profile of childhood pneumonia as a public health priority [6] but much more needs to be done. This study aimed to examine the time, place, and characteristics of the distribution of pneumonia in hospitalized children in the DZH to provide a basis for improving understanding of this issue and to support prevention initiatives.

## Materials and Methods

A descriptive, cross-sectional study was carried out by accessing Health Management Information System (HMIS) register and patient records at DZH from July 16, 2008 to August 17, 2011 for hospitalized children under five years of age and diagnosed with pneumonia based on the International Classification of Diseases 10 (ICD-10) version 2008.

WHO clinical case definition of Pneumonia was used to define Pneumonia. Chest radiographs were taken according to usual clinical practice, which included all children with pneumonia suspected on clinical grounds. All chest radiographs were evaluated by an experienced radiologist. The children who produced sputum were referred to Western Regional Hospital, Pokhara, Nepal for sputum culture to confirm a clinically suspected case of pneumonia.

All patients had fever (temperature more than 38°C) and clinical signs suggesting pneumonia such as dyspnea, fast breathing (child less than two months: 60 breaths per minute; child 2–12 months: more than 50 breaths per minute; child 12–59 months: more than 40 breaths per minute) chest indrawing, and other general danger signs (lethargy, inability to feed, convulsions, vomiting everything).

The authors analyzed the pneumonia-associated hospitalizations by gender, age group, ethnic group, month/season, year, and place using Microsoft Excel 2007. For the ethnic classification, the ethnic categories developed by Health Management Information System (HMIS), DoHS, MoHP (Table 1) were used.

**Table 1:** Ethnic codes as defined by the Health Management Information System.

Ethnic Codes	Ethnic Categories	Ethnics
1	Dalit	Hills of Kami, Damai, Sharki, Gaine, Badi
2	Disadvantaged Janajatis	Hills of Magar, Tamang, Rai, Limbu, Sherpa, Bhote, Walung, Sunuwar, Kumal, Jirel, Danuwar, Thami, Raji
3	Disadvantaged non Dalit Terai caste groups	Yadav, Teli
4	Religious minorities	Muslims, Chureto
5	Relatively advantaged Janajatis	Newar, Thakali, Gurung
6	Upper caste groups	Brahmin, Chhetri, Thakuri, Sanyashi, Raajput, Kayastha, Baniya, Marwadi, Jaire, Nurang, Bengali

Ethical approval was specifically waived by the Pokhara University Institutional Review Board as it does not involve direct human participants. The written request to collect data of the hospitalized patients was made by the researchers on behalf of Pokhara University which was accepted by Head of Department of Dhaulagiri Zonal Hospital.

## Results

A total of 772 children under five years were diagnosed with pneumonia from July 16, 2008 to August 17, 2011 at DZH. The main bacterial causes of pneumonia were *Streptococcus pneumoniae* and Haemophilus influenzae type b (Hib), and the main viral cause was Respiratory Syntical Virus (RSV). *Streptococcus pneumoniae* was confirmed in 122 children (15.8% overall, Table 2). Respiratory Syntical Virus (RSV) was the most common viruses being identified in 97 (12.6%) cases. Influenza A was second in frequency (7.5%) followed by Parainfluenzae type 3 (4.0%), and influenza B (3.5%). In addition, 19% of the cases were of mixed etiology i.e., cases that fulfilled the etiologic diagnostic criteria described above for more than one pathogen).

**Table 2:** Microorganisms identified in children under five hospitalized for pneumonia (n = 772).

Microorganisms	No. of children (%)
*Streptococcus pneumoniae*	122 (15.8)
Haemophilus influenzae type b (Hib)	71 (9.2)
Respiratory Syntical Virus (RSV)	97 (12.6)
Influenzae A	58 (7.5)
Parainfluenzae type 3	31 (4.0)
Influenzae B	27 (3.5)
Mixed pneumonia	147 (19.0)
Pneumonia but not pathogen identified	219 (28.4)
Total	772

**Figure 1:**
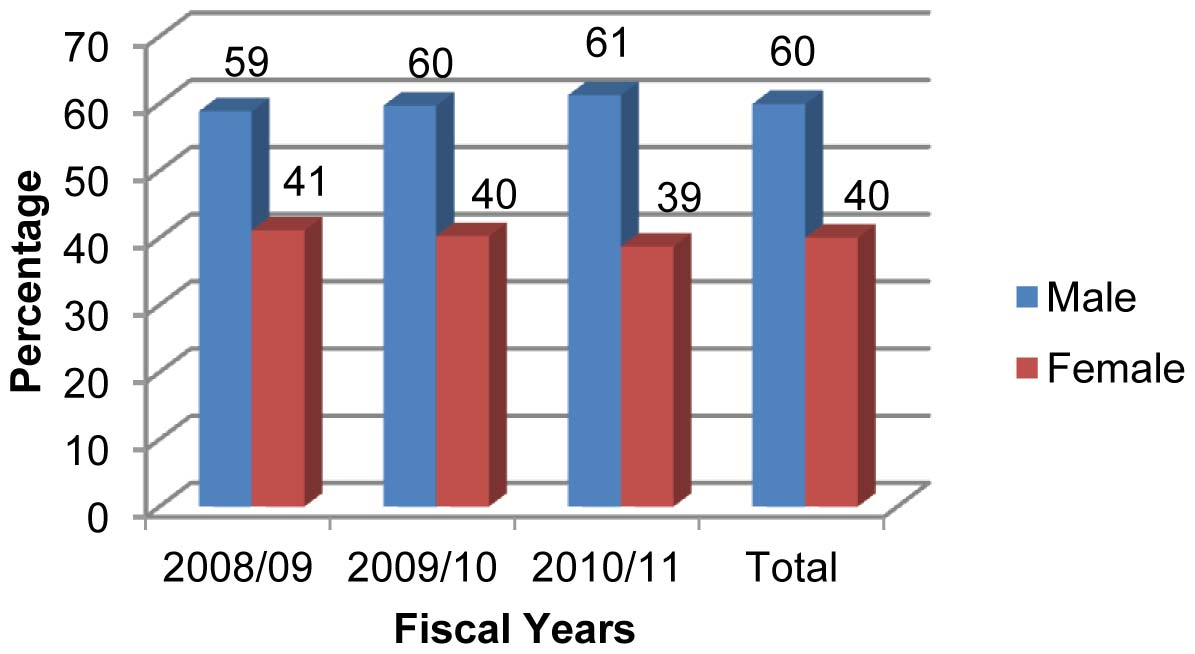
Gender distribution of children under five with pneumonia.

**Figure 2:**
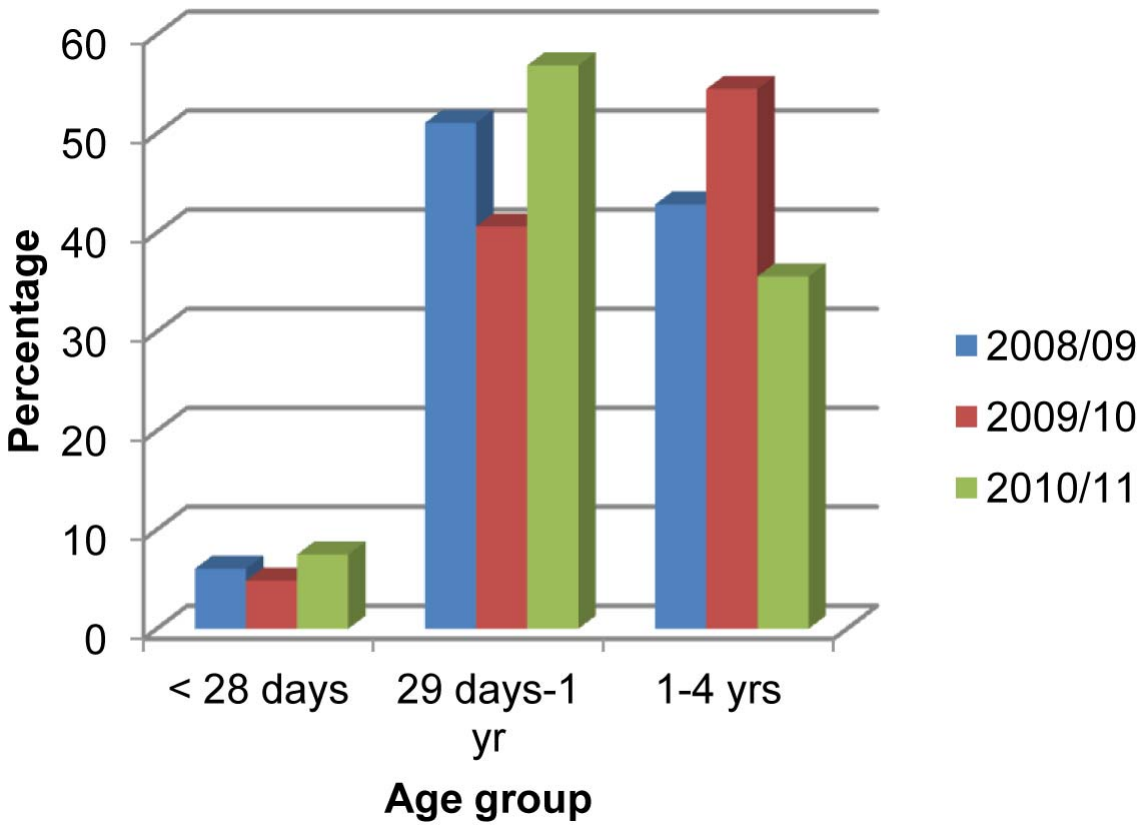
Age distributions of children under five with pneumonia.

**Figure 3:**
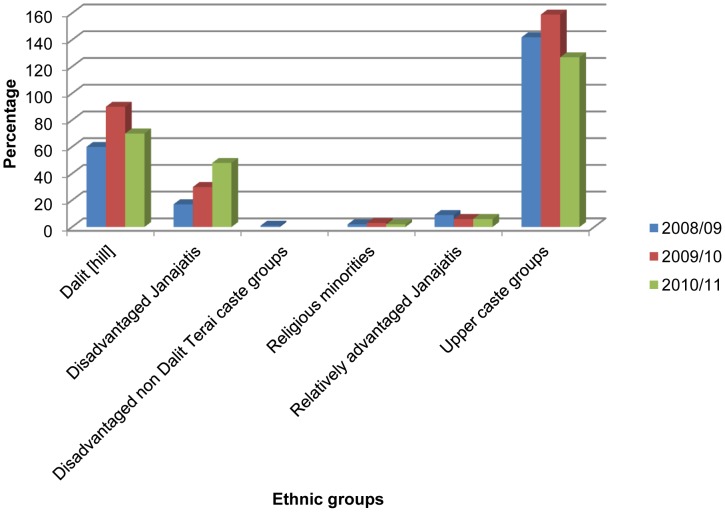
Ethnic distributions of children under five with pneumonia.

Of these children, majority 463 (60%) were males (Figure 1). Forty nine per cent of pneumonia cases (379) were found among those aged 29 days to one year followed by 1–4 years of age (346, 44.8%) and the lowest incidence (47, 6.1%) was found in neonates (those under 28 days of age) (Figure 2). Among the ethnic groups, the highest proportion of children with pneumonia were of the upper caste (having a comparative advantage in terms of social and economic status), followed by the Dalit (a socioeconomically disadvantaged group in Nepal) and Janajati (an indigenous disadvantaged group) in each year (Figure 3).

**Figure 4:**
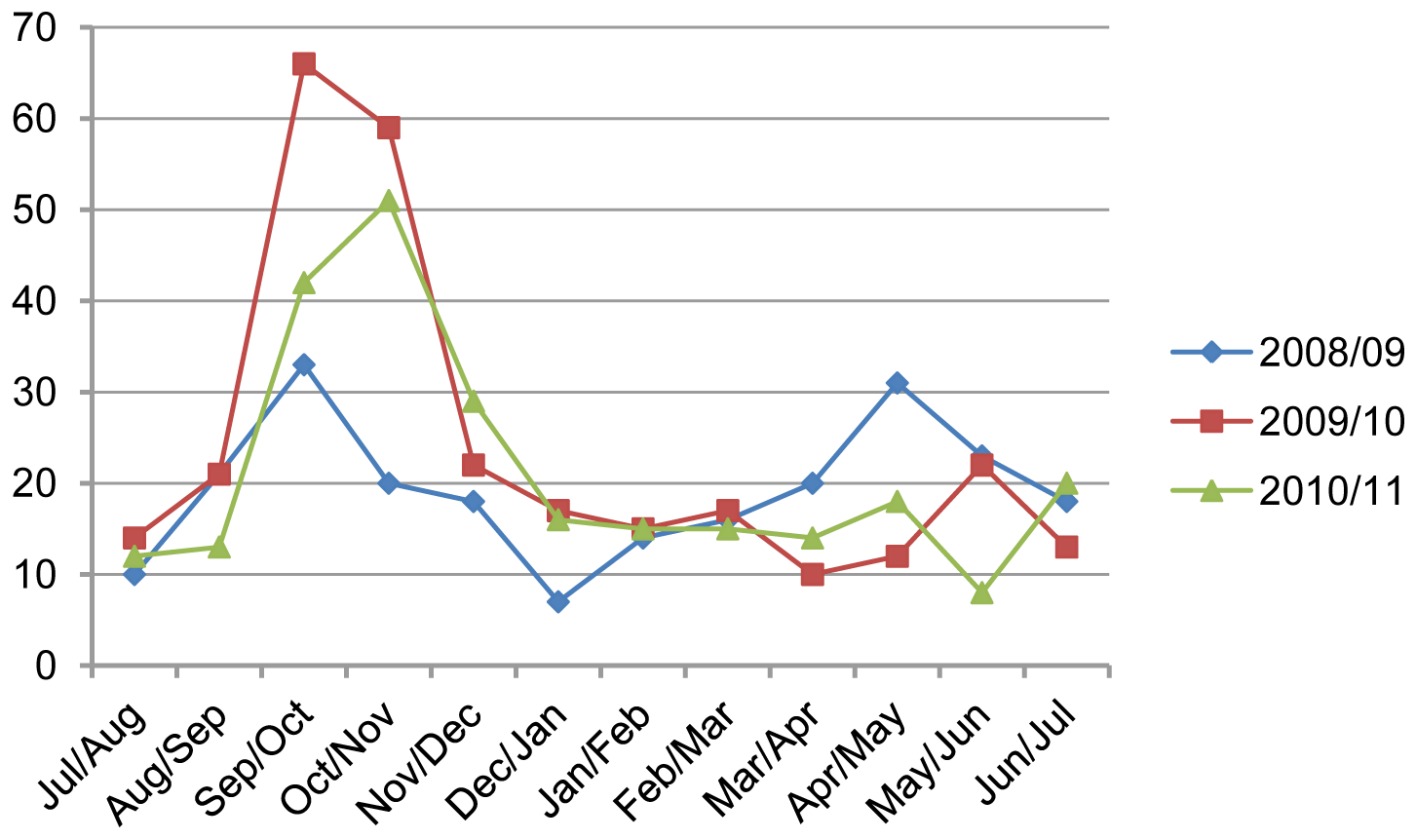
Monthly distributions of children under five with pneumonia.

Pneumonia episodes occurred throughout the year with a sharp increase in the occurrence at the end of August to September (Figure 4). There were also significant pneumonia incidents at the end of March to April in the years 2008/09 and 2010/11. However, in the year 2009/10 smaller peaks of pneumonia were found in May. More than 50% cases were seen during the last five months of all years except for the year 2008/09. More cases were recorded during the rainy seasons and in winter months in all three study years.

All children under five with pneumonia are around 6% of total in-patient department cases. In the three consecutive years, the percentage share of children under five with pneumonia in the in-patient department was highest (7.11%) in the year 2009/10 (Table 3).

**Table 3:** Proportion of total inpatient admissions of children under five for pneumonia.

SN	Year	Total In-patient Department Cases	Total Pneumonia cases	Total U5* pneumonia cases	%age of pneumonia in U5 children among total cases of pneumonia	%age of U5 pneumonia among all hospitalized patients
1	2008/09	5126	311	231	74.3	4.51
2	2009/10	4048	384	288	75.0	7.11
3	2010/11	3805	336	253	75.3	6.65
	Total	12979	1031	772	74.9	5.95

* Under five.

Most of the cases (31%) were from the Baglung municipality followed by its surrounding district Parbat (10.2%) and from Tityang (7.1%), Bhakunde (4%), and Bhimpokhara (3.6%).

## Discussion

A descriptive analysis of seven hundred seventy two cases of children under five years hospitalized with pneumonia in DZH was conducted to elucidate the epidemiology of childhood pneumonia in the Baglung district, as well as in the Dhaulagiri zone of Nepal. The study identified a significant proportion of the under five pneumonia burden as being borne by male (six male sufferers for every ten cases of under five pneumonia) with the male female ratio of 1.5∶1. A study conducted on children hospitalized with pneumonia in Taiwan showed male and female ratio as 1.27∶1 [8]. The same finding of health disparity being associated with higher pneumonia cases common among hospitalized male children then in female has been reported by the studies conducted in Bangladesh [9], [10] where the male female ratio was 2∶1 and 1.4∶1 respectively. It may be because male children are more vulnerable to pneumonia and because they are given more care in our society than female children.

Studies conducted in Taiwan [8] and Bangladesh [10] have shown that the annual age-wise distribution of children with pneumonia is the highest in infants, which is consistent with the present study. This is possibly because they are more susceptible to viral and general infections [11]–[13].

The percentage of pneumonia was found to be highest in children from upper ethnic group, followed by Dalit and the disadvantaged Janajati. Factors such as greater population density, higher service utilization and awareness level, and income among the upper ethnic groups substantially increased the case.

Pneumonia was more common during the rainy seasons and winter months, reaching peak in early September. The rise in pneumonia cases in September is usual as WHO [2] stated that the peaks typically occur in the rainy season in tropical climates, and Baglung has this kind of weather. This finding is similar to other pneumonia study conducted in Nepal [14].

The majority of the children under five with pneumonia admitted to the hospital were from the Baglung municipality. However, this may not represent a higher occurrence in that area, as this may represent a larger catchment area of the hospital, increased geographical accessibility and higher service utilization among local populations surrounding the Baglung municipality.

This study describes only the distribution of the disease in terms of time, place, and characteristics of the distribution of pneumonia in children under five registered in the in-patient department of DZH. There is a need for more research to identify the cases other than those hospitalized in DZH and factors underlying the condition.

## Conclusions

Pneumonia requiring hospitalization is more common among male children, those aged 29 days to one year, and in upper ethnic groups, during the rainy seasons and in winter months, and among residents of Baglung municipality where the hospital is located. The findings of this study will be useful for the policy makers, health workers, stakeholders, and all those associated with the disease to better understand the situation of pneumonia among children under five years in Baglung and its surrounding districts. Moreover, the findings provide reference data for planning, organizing, and evaluation of childhood pneumonia control program in these areas. Strengthening community-based case management, prevention strategies and health care delivery system will help reduce pneumonia cases and the overall burden associated with it.
